# Methylamine-Sensitive Amperometric Biosensor Based on (His)_**6**_-Tagged *Hansenula polymorpha* Methylamine Oxidase Immobilized on the Gold Nanoparticles

**DOI:** 10.1155/2014/480498

**Published:** 2014-07-16

**Authors:** Nataliya Ye. Stasyuk, Oleh V. Smutok, Andriy E. Zakalskiy, Oksana M. Zakalska, Mykhailo V. Gonchar

**Affiliations:** ^1^Institute of Cell Biology, NAS of Ukraine, Drahomanov Street 14/16, Lviv 79005, Ukraine; ^2^Institute of Applied Biotechnology and Basic Sciences, University of Rzeszow, Sokolowska Street 26, 36-100 Kolbuszowa, Poland

## Abstract

A novel methylamine-selective amperometric bienzyme biosensor based on recombinant primary amine oxidase isolated from the recombinant yeast strain *Saccharomyces cerevisiae* and commercial horseradish peroxidase is described. Two amine oxidase preparations were used: free enzyme (AMO) and covalently immobilized on the surface of gold nanoparticles (AMO-nAu). Some bioanalytical parameters (sensitivity, selectivity, and storage stability) of the developed biosensors were investigated. The sensitivity for both sensors is high: 1450 ± 113 and 700 ± 30 A^−1^ *·*M^−1^ *·*m^−2^ for AMO-nAu biosensor, respectively. The biosensors exhibit the linear range from 15 *μ*M to 150 *μ*M (AMO-nAu) and from 15 *μ*M to 60 *μ*M (AMO). The developed biosensor demonstrated a good selectivity toward methylamine (MA) (signal for dimethylamine and trimethylamine is less than 5% and for ethylamine 15% compared to MA output) and reveals a satisfactory storage stability. The constructed amperometric biosensor was used for MA assay in real samples of fish products in comparison with chemical method. The values obtained with both approaches different methods demonstrated a high correlation.

## 1. Introduction

The assay of aliphatic amines in environment and biological samples is important, due to the use of these compounds in industry and their wide distribution in living organisms as a result of natural degradation of proteins, amino acids, and other nitrogen-containing compounds [1]. Methylamine (MA) can be accumulated in some kinds of fish, especially of* Gadoid* species, in very high amounts as a result of enzymatic degradation of the natural osmolyte, trimethylamine* N*-oxide (TMAO). Consuming such fish food in combination with nitrate-containing products could result in the formation of* N*-nitroso derivatives which are very powerful carcinogens [2].

It is also important that MA is widely used in different stages of the preparation of various drug substances; therefore, it may be retained in the drug substance. MA is also controlled by the U.S. Drug Enforcement Administration as a List 1 Regulated Chemical due to its use in production of methamphetamine [3].

It was shown that reactions of trimethylamine (TMA) accumulation are not specific for fish only and may also occur in human organism. This fact has been proven by the discovery of human inherited syndrome of “fish smell,” trimethylaminuria [4]. Homozygous carriers of this metabolic disorder produce a specific smell due to continuous TMA excretion with sweat and urea. This disorder is probably caused by defects in flavoenzyme, monooxygenase FMO3, which converts TMA, a compound with a very unpleasant smell, into TMAO, an odourless compound.

Unfortunately, the problem of analysis of fish products quality and biochemical diagnostics of trimethylaminuria is yet to be solved due to the absence of a fast, cheap, and selective method of analysis of food products and human biological liquids for the presence of primary aliphatic amines, that is, MA, dimethylamine (DMA), and TMA. The majority of the frequently used approaches are time consuming and expensive and require skillful labor techniques, such as the high performance liquid chromatography [5], colorimetry [6], laser spectroscopy [7], fluorometry [8], ion chromatography [9], and enzymatic method [10]. The majority of these methods are marked by poor precision and low sensitivity and selectivity.

The simplest way to detect alkylamines is an enzymatic method based on natural isoforms of amine oxidases with different substrate specificity. The main obstacle for using this method is absence of commercial preparations of primary amine oxidase (AMO; E.C. 1.4.3.21), the key biologically active element of a potential biosensor.

In this paper, we describe the construction of an AMO-based amperometric bioelectrode selective to MA. A ferrocene layer was used as a final electron transfer mediator. The biorecognition membrane includes two enzymes: purified (His)_6_-tagged AMO from the recombinant yeast* Saccharomyces cerevisiae* [11] and commercial horseradish peroxidase (HRP). The enzymes were dropped on the ferrocene-modified graphite electrode surface and covered with a cathodic polymer GY 83-0270 0005. The principal scheme of MA determination is as follows:

(1) CH3NH2+H2O+O2→AMOCH2O+NH3+H2O2,H2O2+HRP(Fe)⟶H2O+HRP(Fe=O),HRP(Fe=O)+ferrocene(Fe2+)+2H+⟶HRP(Fe)  +ferrocene(Fe3+)+H2O,ferrocene(Fe3+)→Electrodeferrocene(Fe2+)

The developed amperometric bienzyme electrode was applied for MA assay in the samples of fish products.

## 2. Materials and Methods

### 2.1. Materials

Horseradish peroxidase (HRP) (EC 1.11.1.7, from* Armoracia rusticana*, 500 U*·*mg^−1^), tetrachloroauric acid trihydrate, sodium salt of ABTS, sodium citrate,* N*-(3-dimethylaminopropyl)-*N*′-ethylcarbodiimide hydrochloride (EDC), pentafluorophenol (PFP), 2-(2-aminoethoxy) ethanol (AEE), dimethylformamide (DMF),* N*,*N*-diisopropylethylamine (DIPEA), 16-mercaptohexadecanoic acid (MHDA), methylamine (MA), dimethylamine (DMA), trimethylamine (TMA), NaCl, KH_2_PO_4_, Na_2_HPO_4_, chloroform, and Butvar solution B-98 were purchased from Sigma-Aldrich (ALSI Ltd., Kiev, Ukraine). The cathodic electrodeposition paint “GY 83–0270 0005” was from BASF Farben und Lacke (Munster, Germany). All buffers and standard solutions were prepared using the water purified by the Milli-Q system (Millipore).

### 2.2. Isolation and Purification of Amine Oxidase (E.C. 1.4.3.21)

As a source of (His)_6_-tagged AMO, the constructed by us recombinant yeast strain* Saccharomyces cerevisiae* C13ABYS86 (*Mat a, leu2-3, ura3, his, pra1-1, prb1-1, prc1-1, cps1-3*) able to overexpress the target enzyme was used. The (His)_6_-tagged enzyme was purified from the cell-free extract of the recombinant strain by metal-affinity chromatography on Ni-NTA-agarose [11]. Purified AMO with specific activity 13.7 U*·*mg^−1^ was stored in 20 mM phosphate buffer, pH 7.5 (PB), at 4°C.

### 2.3. Assay of AMO Activity

The AMO activity was measured according to the method described by Haywood and Large [10]. AMO activity of free and immobilized on nAu enzyme preparations was determined by monitoring ABTS oxidation by H_2_O_2_ generated during MA cleavage. The millimolar absorption coefficient of the radical-cation product at 405 nm is 18.41 mM^−1^ *·*cm^−1^. Usually, 100 *μ*L of a substrate mixture (150 mM methylamine hydrochloride, 30 mM Na_2_ABTS, and 0.3 mg*·*mL^−1^ HRP in 75 mM PB, pH 7.5) was preincubated for 5 min at 30°C and added to the test tube containing 10 *μ*L of AMO preparation. The reaction mixture was incubated for 15 min at 30°C followed by radical-cation product assay. The kinetic study was carried out at the same conditions within methylamine concentration range of 0.02 to 5 mM. AMO concentration in the incubation mixture was 40 ng*·*mL^−1^. One unit of AMO activity was defined as the amount of enzyme required to catalyse the formation of 1 *μ*mole ABTS radical cation per 1 min under standard conditions.

### 2.4. Synthesis of Gold Nanoparticles (nAu)

Gold nanoparticles (nAu) were synthesized by the citrate reduction method [12]. 1.25 mL of 1 mM HAuCl_4_ and 0.125 mL of 38.8 mM trisodium citrate were mixed at 100°C and stirred for 15 min to obtain a wine-red solution. The nAu were precipitated by centrifugation (6708 g; Hettich Micro-22R centrifuge). The precipitate was washed with water and stored at +4°C before using. Under the described conditions, a colloid solution of nAu in water at concentration of 0.9 mM was obtained for further characterization and enzyme immobilization.

### 2.5. Immobilization of (His)_6_-Tagged AMO on nAu

The nAu were incubated overnight in 5 mM MHDA in ethanol at +4°C. After rinsing with DMF, the MHDA-covered nAu were incubated in a DMF solution of 20 mM CMC, 20 mM PFP, and 20 mM DIPEA for 30 min at 25°C. After repeated rinsing with DMF, condensation of the activated Au-linked carboxylic groups with amine groups of the enzyme was carried out. 25 *μ*L of the enzyme solution (0.75 mg*·*mL^−1^) in 30 mM PB, pH 7.5, was incubated with nAu for 1 h at 25°C. After rinsing with PB, pH 7.5, blocking of unreacted carboxylic groups with 0.1 M solution of AEE in 0.1 M bicarbonate buffer, pH 7.0, was performed. The biofunctionalized nAu were rinsed with PB, pH 7.5, and stored at +4°C until usage. The amount of immobilized enzyme on the nAu was determined as the difference between the initial and unbound protein content in the immobilization medium using the Lowry protein assay method.

### 2.6. Characterisation of nAu Using Atomic Force Microscopy (AFM)

The size and structure of nAu particles (unbound and enzyme-modified) were studied by atomic force microscope Solver P47-PRO (NT-MDT, The Netherlands). An aliquot of the tested sample was spread on the surface of freshly cleaved mica, dried, and analyzed in air using the tapping mode with resonance frequency of 160 kHz, scan rate of 1 Hz/s, and resolution of 256 × 256 pixels.

### 2.7. Apparatus for Biosensor Analysis

Amperometric biosensors were evaluated using constant potential amperometry in a three-electrode configuration with a Ag/AgCl/KCl (3 M) reference electrode and a Pt-wire counter electrode. Amperometric measurements were carried out using a potentiostat CHI 1200A (IJ Cambria Scientific, Burry Port, UK) connected to a personal computer and performed in batch mode under continuous stirring in a standard 40 mL cell at room temperature.

Graphite rods (type RW001, 3.05 mm diameter, Ringsdorff Werke, Bonn, Germany) were used as working electrodes. They were sealed in glass tubes using epoxy glue thus forming disk electrodes. Before sensor preparation, the graphite electrodes were polished with emery paper.

### 2.8. Preparation of the Mediator-Modified Graphite Electrodes

Electrodeposition of 5 mM solutions of Mendola blue and methylene blue in 20 mM PB, *р*Н 7.5, on the surface of 3 mm rod graphite electrode was performed using cyclic voltamperometry in the range from −400 to +400 mV with scan rate 50 mV*·*min^−1^ *versus* Ag/AgCl/3 M KCl reference electrode.

A 5 *μ*L aliquot of 2 mM ferrocene solution in acetone was dropped onto surface of working electrode and air-dried for 10 min.

The mediator-modified electrodes were rinsed with water and equilibrated in 30 mM PB, pH 7.5, before using.

### 2.9. Immobilization of the Enzymes on the Mediator-Modified Electrode Surface

HRP and AMO were dropped on the mediator-modified electrode and covered by an electrodeposited commercial cathode polymer-GY 83-0270 0005 (*CP*). The immobilization procedure was as follows: 3 *μ*L of HRP solution with activity 200 U*·*mL^−1^ in 30 mM PB, pH 7.5, was dropped on the top of the mediator-modified carbon electrode. After drying for 2 min at room temperature, the layer of HRP was covered with a 5 *μ*L solution of AMO in FB, pH 7.5, with activity 40 U*·*mL^−1^ or AMO-nAu, 35.5 U*·*mL^−1^, respectively. Dried bienzyme layers were covered with 4 *μ*L of GY 83-0270 0005 for polymer layer formation. The prepared biofunctionalized electrode was rinsed with PB, pH 7.5, and stored at +4°C before application.

### 2.10. A Colorimetric Method for the Determination of Methylamine in Fish Products

A chemical analysis of MA, based on the use of lactose in alkaline solution [6], was performed using the multiple standard addition method. The absorbance of the reaction product was recorded with a Shimadzu UV-1650 PC spectrophotometer (Shimadzu, Kyoto, Japan) at 545 nm relative to an MA-free control sample. The concentration of MA in the tested samples was determined from a calibration curve.

### 2.11. Preparation of Fish Products Samples

For assay of MA, the frozen sea fish samples of hake and grouper were used. Muscle tissues (12 g and 60 g) of the frozen fillet were ground in a mortar with a following addition of 50 mL of deionized water. Trichloroacetic acid was added to the samples (to the final concentration of 4%). The protein precipitate was separated by passing the resulting mixture through a folded filter. Before analysis, the obtained filtrates were neutralized to pH value 7.5 by 10 M NaOH. The filtrate was stored before analysis at +4°C.

### 2.12. Statistic Treatment

All the measurement's results and the level of correlation between results obtained by the different analytical methods were calculated by computer program Origin 8.0 and Microsoft Excel.

## 3. Results and Discussion

### 3.1. Evaluation and Optimization of the MA Biosensor

For construction of an MA-selective biosensor, the bienzyme sensor architecture was used which contained (His)_6_-tagged AMO or AMO-modified nAu and commercial HRP. Using an HRP allows decreasing working potential of the sensor to −50 mV which is important to avoid interfering effects of a sample background.

**Figure 1:**
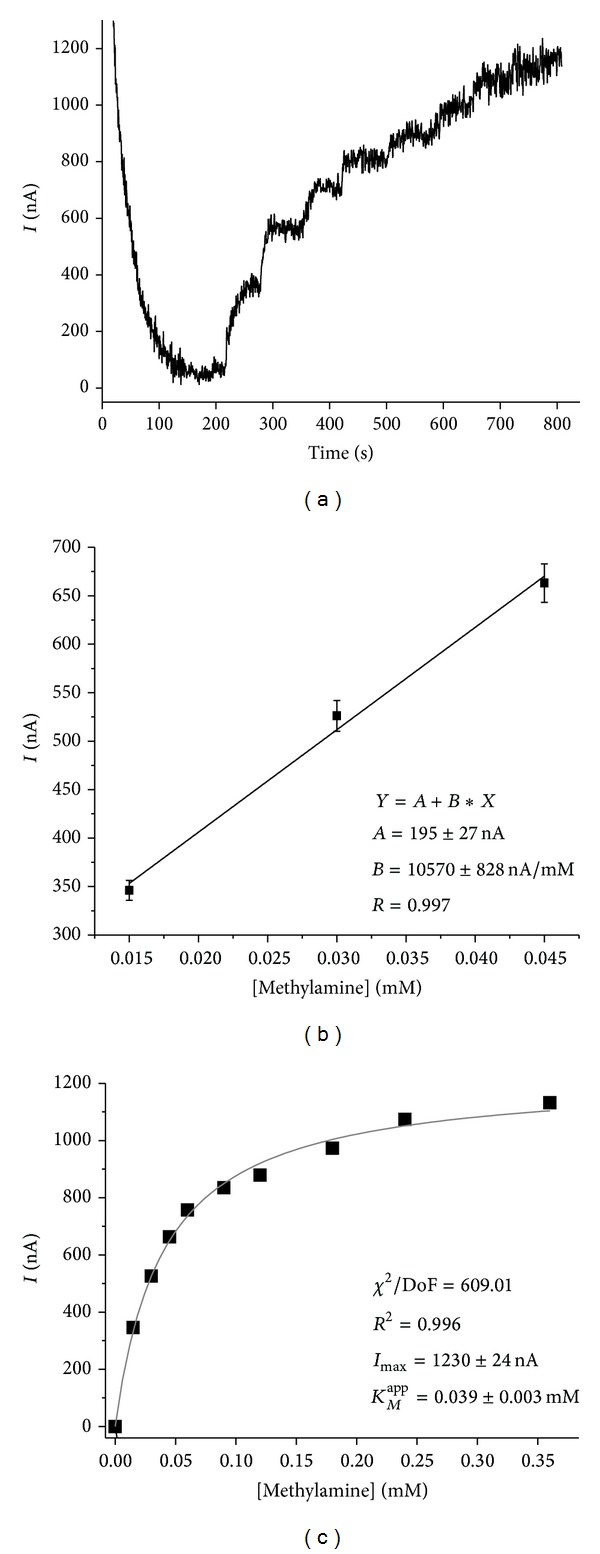
Chronoamperometric current response upon subsequent additions of methylamine (a) and calibration graphs (b), (c) for* CP*-AMO-HRP-ferrocene-modified carbon electrode. Conditions: working potential −50 mV* versus* Ag/AgCl/3 M KCl in 20 mM PB, pH 7.5.

To improve electron transfer from transducer to the HRP, a number of synthetic mediators were tested. Using cyclic voltamperometry, three mediators (ferrocene, Mendola blue, and methylene blue) were analyzed by their ability to increase the effectiveness of electron transfer from the carbon electrode to HRP. The best results were obtained for ferrocene (data not shown), so all the next experiments were performed using ferrocene as electron transfer mediator. For physical fixation of HRP and AMO on the surface of ferrocene-modified carbon electrodes, entrapment of the enzymes in a polymer layer of a cathodic paint GY 83-0270 0005 (*CP*) was used. The optimized sensor architecture which consisted of* CP*-AMO-HRP-ferrocene was used for chronoamperometric investigation of methylamine oxidation (Figure 1).

The maximal current at substrate saturation (*I*_max⁡_) for the* CP*-AMO-HRP-ferrocene-modified carbon electrodes (area 7.3 mm^2^) calculated from calibration graph is 1.23 ± 0.02 *μ*A (Figure 1(c)). The value of *K*_*M*_^app^ to MA derived from the calibration plot for the bienzyme sensor is 0.039 ± 0.003 mM, which is lower compared to *K*_*M*_^app^ of free AMO in solution (0.22 mM) [11]. The sensitivity of the sensor for methylamine is 1450 ± 113 *А* *·*M^−1^ *·*m^−2^ (Figure 1(b)). The linearity of the bioelectrodes is in the range from 15 *μ*M to 60 *μ*M, and the time of response to the target analyte (corresponds to 90% of the output) was found to be about 10 s.

### 3.2. Construction of MA-Selective Bioselective Layer Using nAu

It is known that nanomaterials could improve the sensor characteristics [13]. Biofunctionalisation of synthesized nAu was performed by modification of nAu by 16-mercaptohexadecanoic acid and carbodiimide-pentafluorophenol condensation for covalent binding of the enzyme [14] (see Section 2.5 of Math and Meth.). The activity of AMО during immobilization on the nAu surface is represented in Table 1.

As shown in Table 1, AMO covalently immobilized on the surface of the nAu has kept 59.2 ± 1.2% of the initial enzyme activity.

**Figure 2:**
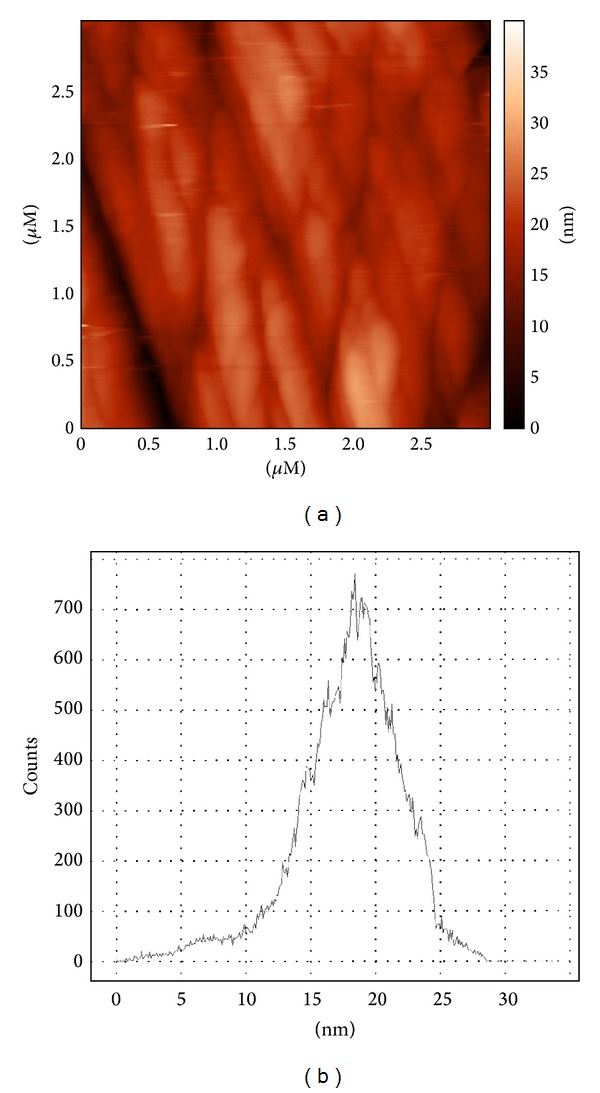
Atomic force microscopy of the AMO-nAu (a) and Gauss distribution of AMO-nAu by size (b).

The Gauss distribution in Figure 2 demonstrates that an average size of the bionanoparticles is close to 17-18 nm.

**Figure 3:**
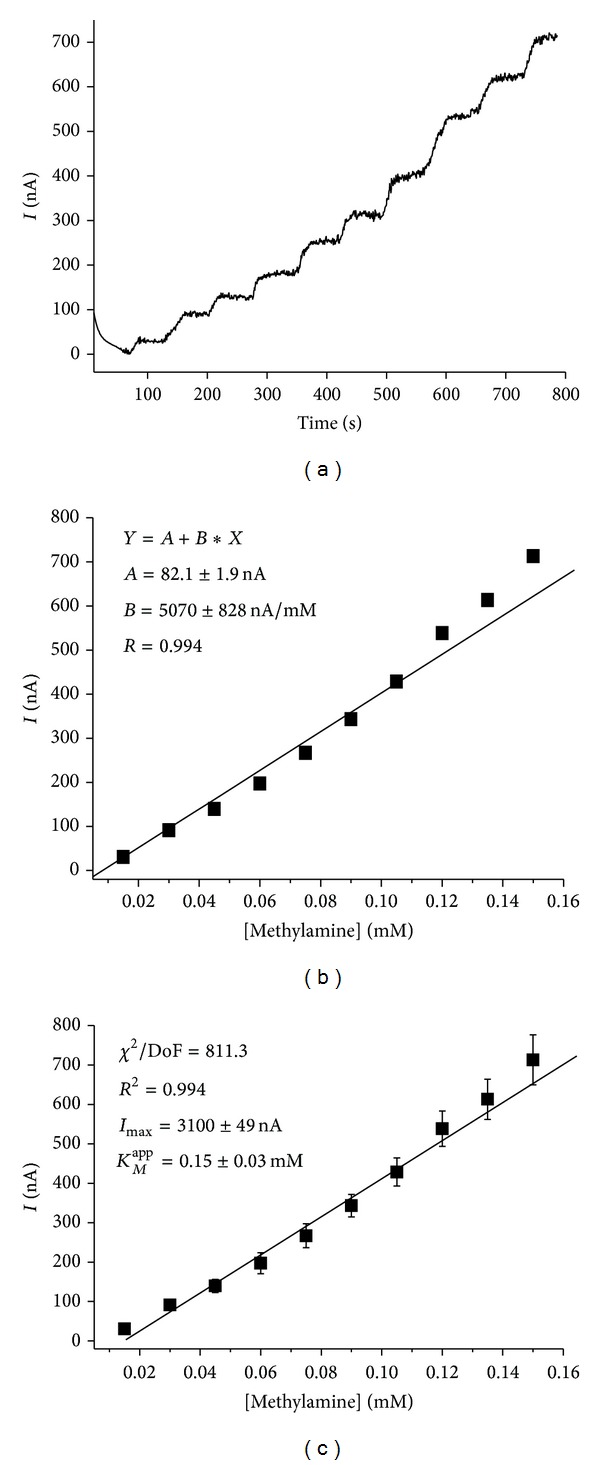
Chronoamperometric current response upon subsequent additions of MA (a) and calibration graphs (b), (c) for* CP*-AMO-nAu-HRP-ferrocene-modified carbon electrode. Conditions: working potential −50 mV* versus* Ag/AgCl/3 M KCl in 20 mM PB, pH 7.5.

Figure 3 represents chronoamperometric current response and calibration curve for the* CP*-AMO-nAu-HRP-ferrocene-modified carbon electrodes upon subsequent additions of MA.

The calibration was performed by a stepwise addition of a standard analyte solution. The sensitivity of the sensor AMO-nAu-based for MA is 700 ± 30 *А* *·*M^−1^ *·*m^−2^ (Figure 3(b)) which is 2-fold lower compared to biosensor without using nAu. The reason for such phenomenon is not clear. The maximal current response of the bioelectrodes modified by nAu (architecture* CP*-AMO-nAu-HRP-ferrocene) calculated from the calibration graph (Figure 3(c)) is equal to 3.10 ± 0.05 *μ*A which is 2.5 times higher compared to* CP*-AMO-HRP-ferrocene sensor architecture (1.23 ± 0.02 *μ*A). The value of *K*_*M*_^app^ to methylamine derived from the calibration plot for the sensor is 0.15 ± 0.03 mM, that is, 3.8 times higher compared to* CP*-AMO-HRP-ferrocene sensor (0.039 ± 0.003 mM). Because of the relatively high affinity of AMO to MA, the observed increased *K*_*M*_^app^ value for AMO-nAu-based electrode has a positive impact on the biosensor properties due to the wider linear range for MA determination (from 0.015 mM up to 0.15 mM). The 2-fold expanded linearity for* CP*-AMO-nAu-HRP-ferrocene sensor architecture is better adapted to the typical concentration range of MA in the real samples. All the next experiments were performed using bioselective layer* CP*-AMO-nAu-HRP-ferrocene architecture.

### 3.3. Assay of MA in Fish Samples

**Figure 4:**
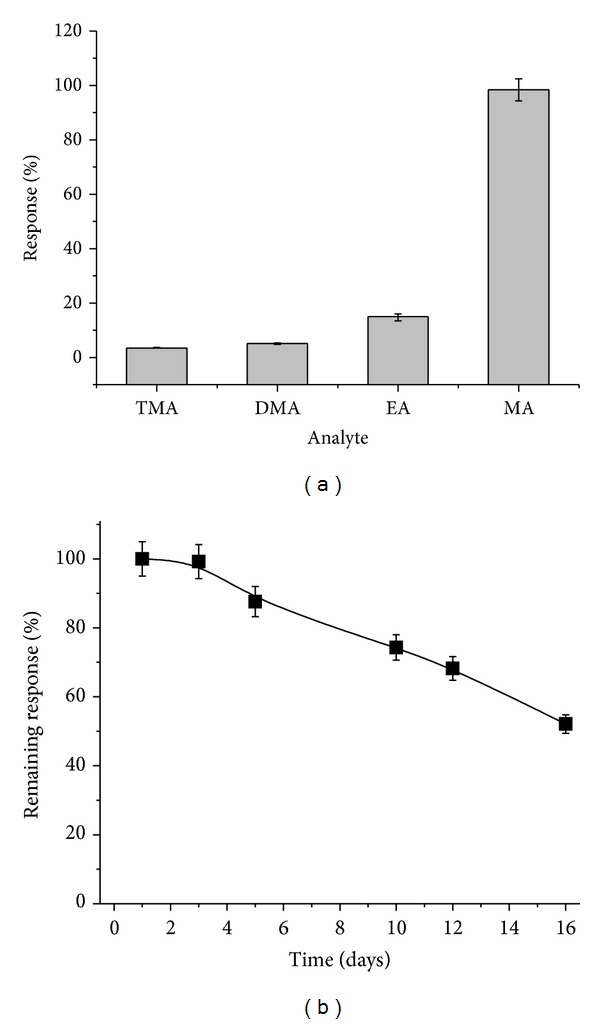
Characteristics of the developed MA biosensor under applied potential of −50 mV at 22°C. The highest current response was chosen as 100% in all experiments. Selectivity test was performed with 0.1 mM solutions of MA, DMA, EA, and TMA in 20 mM PB, pH 7.5 (a); storage stability was checked using 0.05 mM MA in 20 mM PB, pH 7.5, at room temperature during 16 days (b). The bioelectrode was kept at +4°C in 20 mM PB, pH 7.5.

For the application of the constructed biosensor for assay of MA in real samples, the selectivity with respect to structurally similar analytes (DMA and TMA) is of great importance. Hence, the amperometric current response of the developed MA-selective sensor was evaluated with respect to the mentioned above compounds (Figure 4(a)). The storage stability of the sensor was also tested (Figure 4(b)).

As shown in Figure 4(a), positive signals to TMA (3.5 ± 0.17%), DMA (5.1 ± 0.25%), and ethanolamine (EA) (15 ± 1.02%) were detected. It is worth to mention that the possible impact of EA on MA assay is negligible, because this compound (in a free form) is present in fish products only in small amounts (less than 0.019 *μ*g per g) [15]. Thus, the impact of these compounds will not be extremely significant for the correct determination of methylamine in the real samples where the mentioned above components can be present.

For analysis of the storage stability of the sensor, the electrodes were stored between exploitation cycles at +4°C. The measurements were performed at room temperature in 20 mM PB, pH 7.5, with addition of 0.05 mM MA. The half-life of the sensor was close to 16 days of the storage (Figure 4(b)).

**Figure 5:**
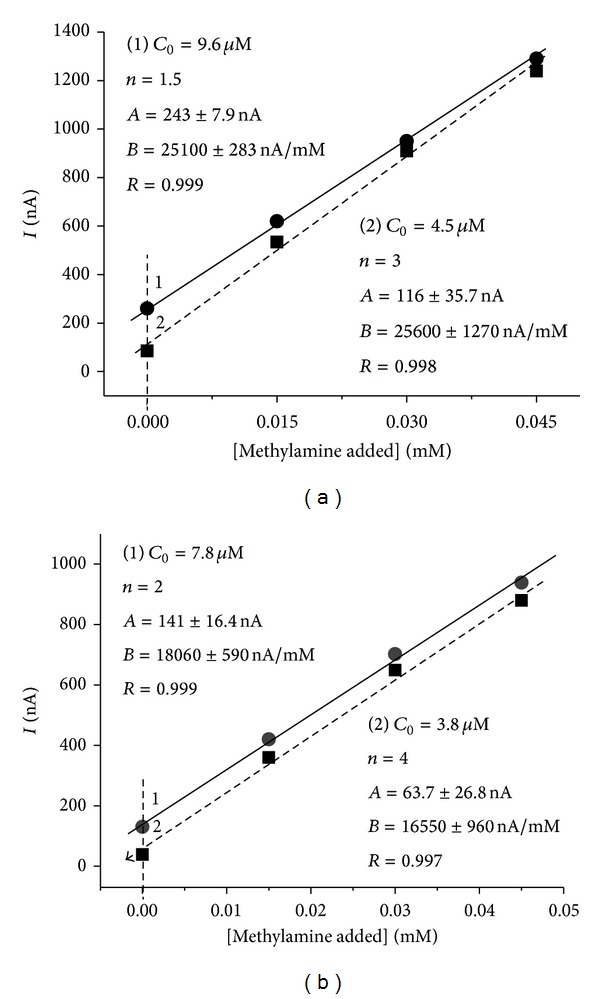
Calibration curves for MA assay in frozen fillet of hake (a) and grouper (b) using standard addition test for biosensor (*CP*-AMO-nAu-HRP-ferrocene).* A*,* B*: parameters for the linear regression line;* n*: dilution factor; *С*_o_: calculated initial concentration;* R*: correlation coefficient.

In order to demonstrate the practical feasibility of the constructed biosensor with architecture* CP*-AMO-nAu-HRP-ferrocene, MA content in frozen fillet of hake and grouper was determined, using standard addition method (Figure 5).

The content of MA in frozen sea fish samples calculated from the calibration curves for standard addition test using MA-selective biosensor was 14.0 ± 0.5 *μ* *М* for fillet of hake and 15.4 ± 0.2 *μ* *М* for grouper, respectively.

The results of MA analysis in the real samples by the developed biosensor were compared with the results obtained by chemical method (Table 2).

Only slight differences between the results obtained by biosensor's approach and chemical method were observed (2.1–2.5%). Moreover, the obtained MA contents results are in good correlation with literature data for sea fish samples [16]. These results demonstrate the perspectives for application of the developed* CP*-AMO-nAu-HRP-ferrocene-based electrode for a correct analysis of MA in food technology.

## 4. Conclusions 

A new methylamine-sensitive bienzyme amperometric biosensor based on the recombinant methylamine oxidase and commercial horseradish peroxidase has been developed. To improve sensor characteristics, covalent immobilization of AMO on the surface of nAu has been performed. The main sensor characteristics (sensitivity, linearity, response time, and *K*_*M*_ value) have been investigated. Testing MA-sensitive laboratory prototype of biosensor on the real samples of sea fish using the multiple standard addition method has been done.

## Figures and Tables

**Table 1:** The activity of AMO during immobilization on the nAu surfaces.

	Enzyme preparation	Supernatant after enzyme immobilization	Immobilized AMO on nAu
*V*, mL	0.03	0.015	0.02
*C*_protein_, mg*·*mL^−1^	0.75	0.52	∗
Total_protein_, mg	0.02	0.08	∗
*A*, U*·*mL^−1^	40.1 ± 1.1	2.55 ± 0.12	35.5 ± 0.2
Total *A*, U	1.2 ± 0.1	0.04 ± 0.002	0.71 ± 0.01
Yield (%)	=100	3.3 ± 0.1	59.2 ± 1.2

*Under sensitivity.

**Table 2:** MA content in frozen sea fish determined by different methods.

Samples	Methods				Difference, %
	MA-selective biosensor		Chemical method, *μ* *М*	
	*μ* *М* (in extract)	*μ*g/g (in wet meat)	*μ* *М* (in extract)	*μ*g/g (in wet meat)
Hake	14.0 ± 0.5	2.4 ± 0.1	13.7 ± 1.2	2.3 ± 0.1	2.1
Grouper	15.4 ± 0.2	2.6 ± 0.1	15.8 ± 0.3	2.7 ± 0.2	2.5
